# Internet-Based Social Connections of Black American College Students in Pre–COVID-19 and Peri–COVID-19 Pandemic Periods: Network Analysis

**DOI:** 10.2196/55531

**Published:** 2024-10-28

**Authors:** Eun Lee, Heejun Kim, Yildiz Esener, Terika McCall

**Affiliations:** 1 Department of Scientific Computing Pukyong National University Busan Republic of Korea; 2 Department of Information Science University of North Texas Denton, TX United States; 3 Section of Biomedical Informatics and Data Science Yale School of Medicine New Haven, CT United States; 4 Division of Health Informatics, Department of Biostatistics Yale School of Public Health New Haven, CT United States; 5 Center for Interdisciplinary Research on AIDS Yale School of Public Health New Haven, CT United States

**Keywords:** COVID-19 pandemic, college students, Black American, African American, social network analysis, social media, mental health, depression

## Abstract

**Background:**

A global-scale pandemic, such as the COVID-19 pandemic, greatly impacted communities of color. Moreover, physical distancing recommendations during the height of the COVID-19 pandemic negatively affected people’s sense of social connection, especially among young individuals. More research is needed on the use of social media and communication about depression, with a specific focus on young Black Americans.

**Objective:**

This paper aims to examine whether there are any differences in social-networking characteristics before and during the pandemic periods (ie, pre–COVID-19 pandemic vs peri–COVID-19 pandemic) among the students of historically black colleges and universities (HBCUs). For the study, the researchers focus on the students who have posted a depression-related tweet or have retweeted such posts on their timeline and also those who have not made such tweets. This is done to understand the collective patterns of both groups.

**Methods:**

This paper analyzed the social networks on Twitter (currently known as X; X Corp) of HBCU students through comparing pre–COVID-19 and peri–COVID-19 pandemic data. The researchers quantified the structural properties, such as reciprocity, homophily, and communities, to test the differences in internet-based socializing patterns between the depression-related and non–depression related groups for the 2 periods.

**Results:**

During the COVID-19 pandemic period, the group with depression-related tweets saw an increase in internet-based friendships, with the average number of friends rising from 1194 (SD 528.14) to 1371 (SD 824.61; *P*<.001). Their mutual relationships strengthened (reciprocity: 0.78-0.8; *P*=.01), and they showed higher assortativity with other depression-related group members (0.6-0.7; *P*<.001). In a network with only HBCU students, internet-based and physical affiliation memberships aligned closely during the peri–COVID-19 pandemic period, with membership entropy decreasing from 1.0 to 0.5. While users without depression-related tweets engaged more on the internet with other users who shared physical affiliations, those who posted depression-related tweets maintained consistent entropy levels (modularity: 0.75-0.76). Compared with randomized networks before and during the COVID-19 pandemic (*P*<.001), the users also exhibited high homophily with other members who posted depression-related tweets.

**Conclusions:**

The findings of this study provided insight into the social media activities of HBCU students’ social networks and communication about depression on social media. Future social media interventions focused on the mental health of Black college students may focus on providing resources to students who communicate about depression. Efforts aimed at providing relevant resources and information to internet-based communities that share institutional affiliation may enhance access to social support, particularly for those who may not proactively seek assistance. This approach may contribute to increased social support for individuals within these communities, especially those with a limited social capacity.

## Introduction

### Overview

The COVID-19 pandemic significantly impacted the mental health of college students [1]. Reports suggest that symptoms of depression, anxiety, and stress increased during the COVID-19 pandemic period [2]. Many factors that were exacerbated by the COVID-19 pandemic, such as housing, food insecurity, and physical isolation, may have negatively affected the mental health of individuals [1,2]. In addition, racial and ethnic minoritized communities were disproportionately impacted by the higher rates of COVID-19 infection and mortality in the United States [3-6]. Owing to structural health inequities, Black American college students are more likely to experience difficulties in accessing resources for basic needs, which may lead to deleterious mental health symptoms [7].

Expanding upon our previous study [8], which mainly focused on the differences in the internet-based conversation topics of the students of historically black colleges and universities (HBCUs; defined as “...any historically Black college or university established before 1964, with the principal mission of educating Black Americans...” [9]) depending on their depression expression using topic analysis and network analysis, this study shifts its focus to examine changes in internet-based social-connection patterns among 2 groups of HBCU students before and during the COVID-19 pandemic. The study includes (1) a depression-related group (d-r group), who have posted a depression-related tweet or a retweet in the pre–COVID-19 pandemic period, and (2) a non–d-r group, who have not posted depression-related tweets. We examined the changes in the 2 groups’ internet-based social networks and investigated the social connectedness of this minoritized group based on whether each Twitter (currently known as X; X Corp) user had a history of mentioning depression. The pre–COVID-19 characteristics of their social networks, such as their mutuality, the extent of the clustering, and other structural properties, have been compared with the same characteristics during the peri–COVID-19 period. This was done to understand whether the aforementioned social network measures have changed during the COVID-19 pandemic or not and the implications of these findings.

### Background

A global-scale pandemic, such as COVID-19, can greatly impact society and result in changes in personal social network structures. The physical restrictions imposed during the COVID-19 pandemic limited face-to-face interactions, which facilitated the expansion of their internet-based social networks [10]. For example, local area–based and internet-based networks emerged in the United Kingdom due to geographic restrictions (eg, stay-at-home orders) for regional volunteers and neighboring supports [11]. Limited social activities and communication, which may lower one’s sense of social connection, can negatively affect mental health. People have used internet-based social platforms to fulfil their need for connectedness, resulting in a global increase in the average time spent on social media during the COVID-19 pandemic. The average time spent has increased from 54 minutes in 2018 to 65 minutes in 2022 [12,13]. Researchers found that those who engage in these internet-based social activities can experience relief from negative mental-health symptoms of depression, loneliness, and anxiety during the COVID-19 pandemic [14-16]. In particular, this internet-based connectedness can be crucial to minoritized groups during the COVID-19 pandemic. This is crucial not only to attenuate the negative mental effects but also to prompt people to share accurate and up-to-date health-related information [17].

The pandemic also had negative implications for mental health and well-being among the young communities of color. A study found that Generation Z adults (aged 18 to 23 years) were the most stressed generation during the COVID-19 pandemic, reporting increased mood symptoms, stress, and alcohol use during the COVID-19 pandemic [18].

In particular, Black communities experienced significantly higher rates of COVID-19 hospitalization and mortality [3-6,19], which can be attributed to systemic health inequalities, including disenfranchisement from the health care system and escalating mental health risks [20,21]. Acknowledging this unequal impact may correlate with distinct coping strategies for social connections in these communities [22]. Black college students, as younger members of their communities, likely encountered unique challenges due to the swift changes in their academic, occupational, housing, and social environments, exacerbated by the disproportionate effects of the COVID-19 pandemic [19]. The survey study by Molock and Parchem [23] revealed substantial disruptions in various aspects of life of college students of color. These aspects included finances, living situations, academics, and career goals, demonstrating increased stress, anxiety, and depression, along with challenges related to racial injustice among the study participants.

Even though age and racial factors could influence minoritized communities (eg, college students of color) significantly during the COVID-19 pandemic, there is a paucity of research on the impact of the COVID-19 pandemic on their mental health and coping behaviors during this time [23-26]. Some studies investigating the emotional well-being of HBCU students found that it strongly correlates with their social connectedness to others, which is mediated by internet-based social platforms [25]. Researchers have observed that strong social networks are a crucial resource for younger generations to cope with depressive symptoms [27]. This is particularly important for college students in Black communities [25]. While these studies offer valuable insights, they are constrained to conducting individual (ie, egocentric) survey analyses and do not focus on the collective behavioral patterns exhibited by HBCU groups.

### Objectives

To fill this gap, this research focuses on the structural changes in the internet-based social networks of HBCU students to understand the collective internet-based behaviors of the group during the COVID-19 pandemic. By categorizing the students on the basis of their creation and engagement with depression-related content, this study aims to investigate how the aforementioned groups (d-r group and non–d-r group) have shown distinctive behavioral patterns in making friends on the internet and to describe the collective behavioral characteristics among the groups before and during the COVID-19 pandemic.

## Methods

### Overview

Firstly, the researchers selected 20 HBCUs and identified active users who are students of the HBCUs. We separated them into the d-r group and non–d-r group depending on depression-related mentions in their tweets (refer to detailed methods in our previously published paper [8]). Social network data (ie, IDs of followers and friends) of these Twitter users were collected for the pre–COVID-19 and peri–COVID-19 pandemic periods. We compared the changes in the 2 groups’ social networks with a focus on their friends networks during the pre–COVID-19 and peri–COVID-19 pandemic periods. The variations of basic network characteristics such as (1) the number of friends or followers, (2) reciprocity, and (3) assortativity of the d-r group before and during the COVID-19 pandemic were assessed; their statistical differences were tested with the paired 2-tailed *t* test. In addition, we separated the HBCU students’ social networks from other users to focus on the structural changes in the HBCU groups and the differences in structural patterns between d-r and non–d-r groups during the COVID-19 pandemic.

### Data Source and Processing

Initially, we compiled a list of 20 HBCUs with active Twitter followers. To ensure representative sampling, HBCUs were chosen on the basis of their geographical distribution, considering their historical concentration in the Southeastern region owing to past racial segregation practices. Therefore, HBCUs in the Southeast (14/20, 70%), Northeast (3/20, 15%), Southwest (2/20, 10%), and Midwest (1/20, 5%) of the United States were selected.

Subsequently, we identified Twitter users associated with the HBCUs. The examination of tweets pertaining to these educational institutions was conducted by one of the authors (TM), who compiled the list of mentions and hashtags relevant to each HBCU (eg, #school_name, @school_name_ abbreviation). Consequently, we compiled a comprehensive list of users who authored tweets featuring these mentions and hashtags using Twitter’s search application programming interface. We categorized these users into 2 groups: one group (d-r group) mentioning depression-related terms (eg, *depressed, #depressed, depression, #depression, dark place, depressing, #depressing, sad sad #SadSad*) on Twitter and another group (non–d-r group) without such indications.

The first dataset of these users for the pre–COVID-19 pandemic period was collected between May 13, 2019, and May 15, 2019. These data yielded 682 users for the non–d-r group and the same number of users for the d-r group. The second dataset of the same users for the peri–COVID-19 pandemic period was collected between July 19, 2022, and July 21, 2022. This was done to gain insight into the changes in social networks during the COVID-19 pandemic. The peri–COVID-19 pandemic period dataset yielded 508 users for the d-r group (508/682, 74.5% of the d-r group in the pre–COVID-19 pandemic period) and 512 users for the non–d-r group (512/682, 75.1% of the non–d-r group in the pre–COVID-19 pandemic period). There has been a notable decline in user subscriptions to the Twitter platform throughout the COVID-19 pandemic. However, we could track >70% of the previous users in the peri–COVID-19 pandemic period. The datasets encompassed various user attributes such as user ID, account description, and IDs of their followers and friends. In addition, tweet-related attributes, such as tweet ID, were included. Comprehensive details about compiling 20 HBCUs, selecting Twitter users associated with these HBCUs, and separating users into 2 groups are noted in our prior work [8].

### Friendship Network Construction

**Figure 1:**
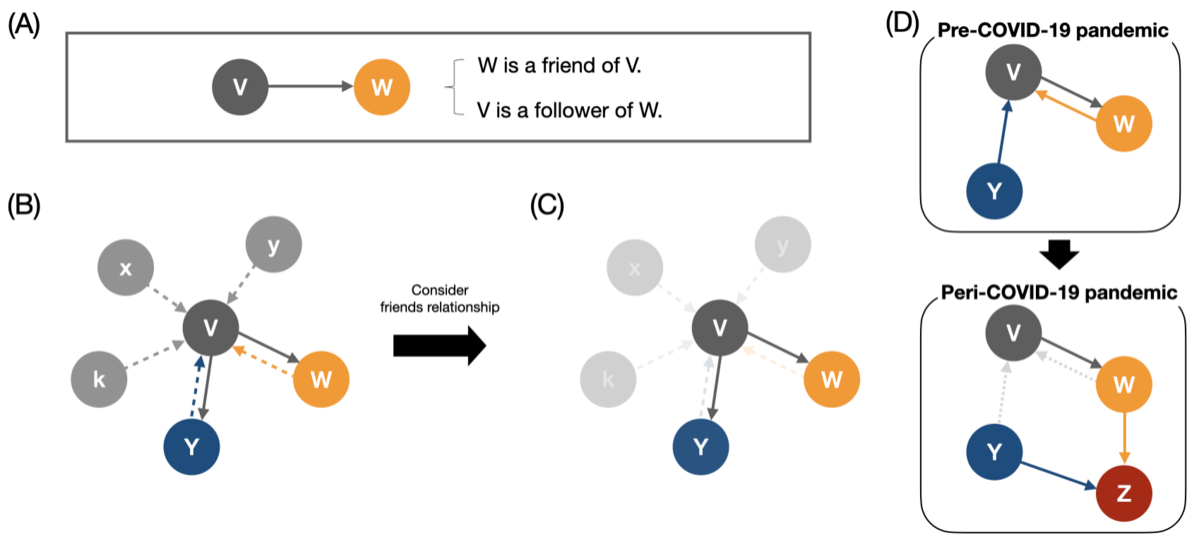
An exemplary friendship network construction from a Twitter account relationship. (A) Two possible interpretations for a relationship between V and W: when V follows W, one can say W is a friend of V or V is a follower of W. (B) A sample social network of person V: dashed arrows represent followers of V, and plain arrows represent the friends of V. Each color follows the originating node’s color. (C) Focusing on V’s friendship network, the internet-based social network can be represented by 3 individuals (V, W, and Y nodes) and their 2 relationships (2 gray arrows). (D) An exemplary network change from pre–COVID-19 to peri–COVID-19 pandemic periods. The pre–COVID-19 network had 3 individuals or nodes V, W, and Y. Then, an individual Z is added to the peri–COVID-19 network, and transparent links between individuals W and V and Y and V disappear in the peri–COVID-19 network.

On Twitter, there are 2 types of relationships: followers and friends. Followers denote people who decided to follow a person’s account; friends are people who an individual account follows, as shown in Figure 1A. We analyzed only “friends” on Twitter as a friendship network because making friends reflects one’s intentional social activities on Twitter, but following relationships are passively decided by others. When V follows W (ie, V→W), W is V’s friend, as shown in Figure 1A. However, V is merely a follower and not a friend of W from W’s perspective. Because we had followers and friends of the sampled HBCU students’ in May 2019 and July 2022, these relationship data were the internet-based social network of HBCU students, including followers and friends, as shown in Figure 1B. By focusing on the friend relationships on Twitter, the network in panel Figure 1B can be transformed to Figure 1C.

From the 2 data collection periods (pre–COVID-19 and peri–COVID-19 pandemic), there were 2 friendship networks for each period; we compared the structural changes of those networks in the context of the COVID-19 pandemic to understand how these 2 groups’ collective internet-based friendships looked different. For instance, Figure 1D displays an exemplary structural change in that W stopped following V and made a new friend, Z. To make a fair comparison, we analyzed the networks of 2019 users solely with those have remained in the 2022 dataset. As the first step, we incorporated all users along with their friends from the collected data to grasp macrolevel patterns. Subsequently, as the second step, we included users only affiliated with the selected HBCUs to explore internal dynamics and microlevel patterns in greater detail.

### The Macroview: Network Analysis With All Users

#### Reciprocity

We defined reciprocity *r_i_* for a node, which bears conceptual resemblance to “mutuality” in sociometry [28] and a network-level “reciprocity” in network science [29]. This was done to quantify the proportion of node i's friends who are in a mutual friendship with node *i*. For instance, let us consider nodes V and W in the upper panel of Figure 1D. Node V has 1 outgoing link to node W (eg, W is a friend of V), and node W has the same number of outgoing links to node V (eg, V is also a friend of W). Because V and W are friends of each other, one can say that there is 1 reciprocal link between nodes V and W. Then, the reciprocity *r_V_* of node V is 1.0 based on equation 1,



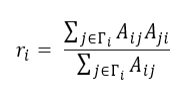



**(1)**

In equation 1, Γ*_i_* denotes a set of i's neighboring friends, and A represents an adjacency matrix of a network. When *A_ij_* = 1, it means there is a link from node i to j, and *A_ij_* = 0 represents a disconnection between node *i* and *j*. Therefore, *r_i_* is in the range of (0-1) by its definition. The larger the reciprocity, the higher the mutual friendship between nodes.

#### Assortativity With the D-R Group of Users

The proportion of the d-r group of users among a node’s friends (eg, a node’s number of outgoing links) could inform us of an individual’s tendency to make friends with the d-r group of users. This type of tendency connecting with similar ones is proposed in many studies [29,30], and in this study, we defined an individual-level “assortativity with the d-r group of users” of node i (*ρ_i_*) as follows:



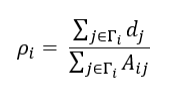



**(2)**

**Figure 2:**
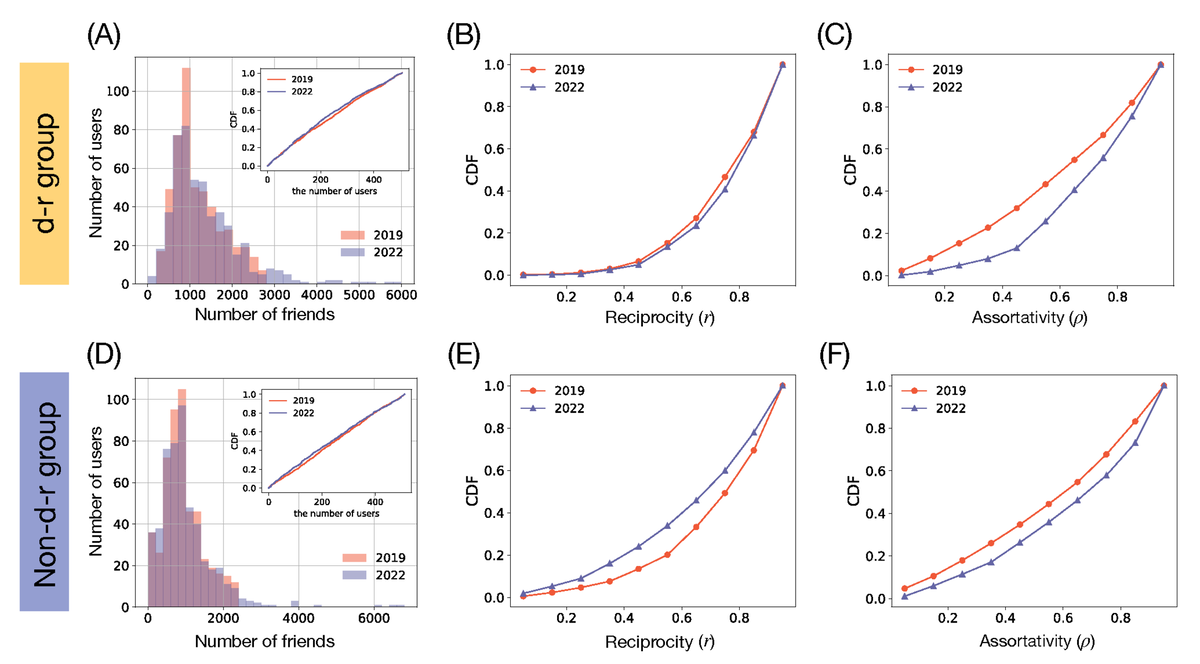
Distributions of the number of friends, reciprocity, and assortativity of the students of historically black colleges and universities with depression-related users (d-r group) and non–depression-related users (non–d-r group). (A-C) The number of friends, reciprocity, and assortativity of the d-r group. (D-F) The number of friends, reciprocity, and assortativity of the non–d-r group. (A and D) Distributions of the number of friends of the d-r group and non–d-r group for pre–COVID-19 (2019; red) and peri–COVID-19 (2022; blue) pandemic periods. The inset shows the cumulative distribution function (CDF) of the number of friends. (B and E) The CDF of the reciprocity (*r*) of the d-r group and non–d-r group for 2019 and 2022. (C and F) The CDF of the assortativity with d-r group (ρ) for each year.

In equation 2, d_j_ denotes a binary value 1 (or 0) when a node is classified as a d-r user in the 2019 dataset (or a non–d-r user in the 2019 dataset). For these 2 network metrics and the number of friends, we used the friendship network for the 2019 and 2022 datasets (as shown in Figure 2).

### Analysis of Networks Among HBCU Students

#### Community Detection

From the friendship network (ie, focusing on the friends’ relationships on Twitter), the largest connected component (LCC) was extracted for each period (pre– or peri–COVID-19 pandemic periods), and the LCC was used for community detection, excluding non-HBCU students. Thus, the size of the LCC for each period was distinct because of different connectivity structures. The LCC of the pre–COVID-19 pandemic period network contains 939 nodes, and the LCC of the peri–COVID-19 pandemic period network includes 827 nodes. We applied the *louvain_communities* function of the *NetworkX* module (Python Software Foundation). This function finds the best partition of a graph based on the Louvain community detection algorithm [31]. Therefore, a node in a network can have the best community membership with an integer number.

#### Membership Entropy

We compared the community membership with the physical affiliations of HBCU students’ accounts. The similarity between a user’s physical affiliation (eg, a university) and their community membership in internet-based network is evaluated as an information entropy, where the fraction of users with a school affiliation in a community is applied as a probability (*p*) in the information entropy equation: *S = –plog (p)*. This entropy quantifies a community’s affiliation complexity in LCC, and we averaged the entropy across all communities as follows:



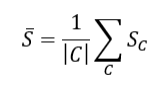



**(3)**

In equation 3, C represents each community, |C| is the total number of communities in a network, and *Sc* denotes the entropy of a community C. The smaller the entropy for a network, the more identical affiliation membership exists in the network’s communities. Putting it differently, the communities in a network are closely matched with the physical affiliation of a college.

#### Coleman Homophily Index

The Coleman homophily index typically ranges from −1 to 1 [32]. A value of 0 indicates a neutral mix of the attributes, representing random connections. A value of 1 signifies complete homophily, where all connections occur within the group; a value of −1 denotes complete heterophily, indicating that all connections with individuals outside the group. When there are 2 groups called *V* and *W* with connections between them, we can assess the homophily of *V* (or *W*) as follows:



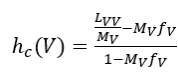



**(4)**

In equation 4, *L_VV_* represents the number of connections between members in group *V*, *M_V_* denotes the total number of connections made by group *V*, and *f_V_* signifies the fraction of group *V*’s size. In this study, the groups are defined as the d-r and non–d-r groups, and *h_c_* illustrates the homogeneity of each group in their internet-based connections during both the pre– and peri–COVID-19 pandemic periods.

#### Attribute Randomized Network Construction

To compare the characteristics of the HBCU students’ friendship network, this research randomized depression-related attributes, which were empirically observed in the LCCs of the friendship network from pre–COVID-19 and peri–COVID-19 pandemic periods. The study generated 100 randomized network samples for each period. The sample networks were structurally identical to the original HBCU students’ friendship networks; however, only depression-related attributes were randomly mixed. To assess the statistical significance of the Coleman homophily index and membership entropy of the randomized networks with those of empirical networks, one can apply the permutation test to those metrics to confirm the statistical significance of their difference.

### Ethical Considerations

To protect the rights and privacy of Twitter users, we followed strict ethical principles throughout the study. All personally identifiable information that could reveal users’ identities was anonymized, and no sample tweet was presented in this paper. Only aggregated information was reported. Data collection, management, and analysis were conducted on a secure research cluster computer. No data were made available as open access.

The University of North Texas (IRB-21-623) and Yale University (2000032441) institutional review boards reviewed and approved the study protocol, determining that it meets the criteria for exemption for research involving human participants. IRB exemptions covered secondary analysis without additional consent for the secondary analyses of this study.

## Results

### The Macroview: Friendship Networks of HBCU Students on Twitter

To understand the HBCU students’ collective internet-based behaviors during the COVID-19 pandemic, first, we compared their friendship networks on Twitter between pre–COVID-19 (May 2019) and peri–COVID-19 (July 2022) pandemic periods, including all users regardless of their affiliation to the sampled HBCUs (Figure 2). In terms of the number of friends, d-r and non–d-r groups’ internet-based friendships have grown on average with time. In particular, the d-r group (with depression-related tweets) expanded the average number of friends from 1194 before the COVID-19 pandemic to 1371 during the COVID-19 pandemic. When we statistically tested the average number of friends for 2 groups using the paired *t* test, the depression-related tweet users (d-r group) had statistically different numbers of friends during the pre–COVID-19 and peri–COVID-19 pandemic periods (*P*<.001), with an increased average number of friends (1194-1371). Conversely, the users without depression-related tweets (non–d-r group) showed a less significant increment (*P*=.06) than the d-r group, even though the former group’s average number of friends slightly increased from 926 to 1007 before and during the COVID-19 pandemic.

The average number of friends and the distribution of the number of friends have changed in the context of the COVID-19 pandemic. The distributions of the number of friends for the d-r group of users in data published in 2019 and 2022 are displayed in Figure 2A. It is noticeable that the tail part stretched out more in 2022 than in 2019, showing the increased number of d-r group of users, with a large number of friends during the COVID-19 pandemic. The heavy-tailed number of friends (approximately 6000) confirms active engagement in making internet-based friends in the d-r group during the COVID-19 pandemic period. The non–d-r group of users’ number of friends also increased in the tail part during the COVID-19 pandemic period (refer to Figure 2D); however, most non–d-r group of users maintained the same number of friends as that before the COVID-19 pandemic, resulting in insignificant difference in the average number of friends (*P*>.05).

Regarding the extent of mutual interactions, the reciprocal relationship between users in both groups displayed statistically significant change during the periods as shown in Table 1 (paired *t* test; d-r group *P*=.01 and non–d-r group *P<*.001) and Figure 2 (for the d-r group, refer to Figure 2B and for the non–d-r group, refer to Figure 2E). However, the pattern of changes in average reciprocity differed between groups. On an average, the d-r group has built up their mutual relationships from 0.78 to 0.8, whereas the non–d-r group has lowered their reciprocity from 0.73 to 0.67, as shown in Figures 2B and 2E). The growth in the d-r group’s reciprocity suggests that users who mention depression on internet-based platforms may be more likely to form mutual relationships on the platform, unlike the non–d-r group.

In addition, the d-r group of users’ assortativity with other d-r group members has been amplified from 0.6 to 0.7 (*P<*.001). Considering that the measure represents the proportion of the d-r group of users when compared with all their friends, it means the d-r group has been connected more to other d-r group of users during the COVID-19 pandemic period (Figure 2C). The non–d-r group of users kept a similar level of assortative connections with the d-r group of users during the period on average; it was not statistically significant (from 0.483 to 0.475, *P*=.62; Table 1 and Figure 2F). The statistically significant increments in the reciprocity (*r*) and the assortativity of the d-r users (*ρ*) show that the d-r group members have made mutual connections with other internet-based users and tend to connect themselves during the COVID-19 pandemic (as seen in Table 1).

**Table 1:** Paired *t* test to measure changes in the number of friends, reciprocity, and assortativity between the pre–COVID-19 and peri–COVID-19 pandemic periods for the depression-related (d-r) and non–depression-related (non–d-r) groups (with their *P* value regarding the paired *t* test).

Measures		Pre–COVID-19 pandemic period (May 2019)	Peri–COVID-19 pandemic period (July 2022)	*P* value
**Non**–**d-r group**				
	Number of friends	926	1007	.06
	Reciprocity	0.73	0.67	<.001
	Assortativity	0.483	0.475	.62
**D-r group**				
	Number of friends	1194	1371	<.001
	Reciprocity	0.78	0.8	.01
	Assortativity	0.6	0.7	<.001

### The Microview 1: Connection Patterns of HBCU Students’ Friendship Networks

**Figure 3:**
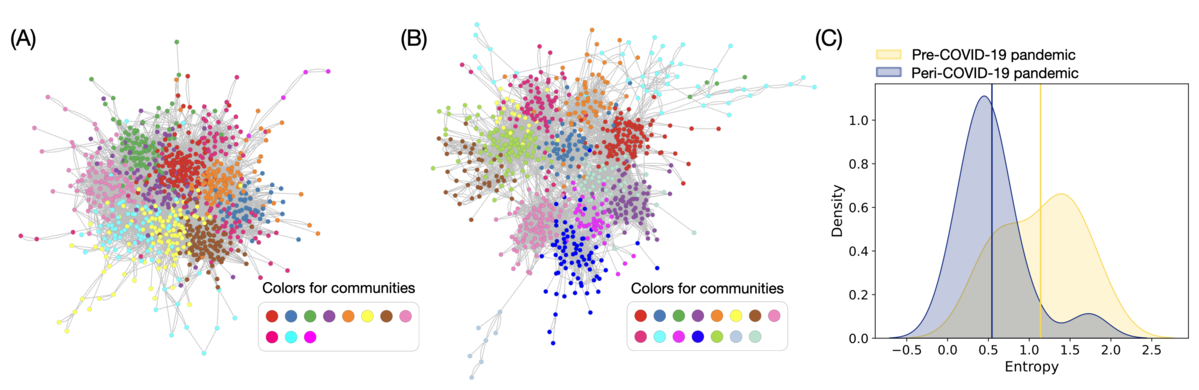
Detected communities in the internet-based social networks of the students from historically black colleges and universities during pre–COVID-19 and peri–COVID-19 pandemic periods. (A) This panel shows communities in the pre–COVID-19 pandemic period (data from May 2019). The colors represent 11 different communities detected by the Louvain community detection algorithm (Python Software Foundation). (B) The panel shows communities in the peri–COVID-19 pandemic period (data from July 2022), which are distinguished by 15 communities. (C) The kernel density estimation plot of the detected communities’ group membership entropy is shown. The vertical lines display the average membership entropy for each period (pre–COVID-19: yellow; peri–COVID-19: blue), and the networks’ layouts use Cytoscape program’s Prefuse Force Directed Layout with default parameter options.

The friendship networks used in the previous analysis included Twitter users who were not identified as HBCU students. Therefore, focusing on HBCU students’ internet-based behaviors could be difficult. Hence, to observe specific characteristics of the HBCU communities, we separated their network from the whole network. After removing users who not identified as HBCU students from the previous LCC, we extracted the largest connected cluster that can represent most of the HBCU students’ network and its connection properties. In the largest connected cluster that connects solely HBCU students, the remaining pre–COVID-19 and peri–COVID-19 pandemic networks included 939 and 827 HBCU students, respectively, including the d-r and non–d-r groups of users (Figures 3A and 3B). These numbers correspond to about 68.8% (939/1364) and 81.1% (827/1020) of the HBCU student sample in the pre–COVID-19 and peri–COVID-19 pandemic periods.

The communities are shown in Figures 3A and 3B, and those communities are determined by the Louvain algorithm [31] in Python’s NetworkX package. This algorithm found 11 and 15 communities for pre–COVID-19 (Figure 3A) and peri–COVID-19 (Figure 3B) pandemic networks, respectively. It is noticeable that the peri–COVID-19 pandemic network is more segregated than the pre–COVID-19 pandemic network even though we displayed the network with the same parameters and layout algorithm (Cytoscape’s Prefuse Force Directed Layout with default options), and the large numbers of communities for peri–COVID-19 pandemic confirm the different segregation (ie, 11 for pre–COVID-19 and 15 for peri–COVID-19 pandemic periods; Figure 3B). The increase of the clustering in the peri–COVID-19 pandemic period is also confirmed by the modularity, a metric of the segregation quality [33]. The pre–COVID-19 pandemic network has a modularity of 0.7; the peri–COVID-19 pandemic has a higher modularity of 0.75, confirming a more distinct and internally cohesive structure of the peri–COVID-19 pandemic network.

The pre–COVID-19 pandemic network has a shorter diameter of 11 (the longest distance between 2 nodes in an entire network) than the peri–COVID-19 pandemic one (diameter of 13). This is because the pre–COVID-19 pandemic network is more densely connected among the communities. The longer diameter in the peri–COVID-19 pandemic friendship network implies that the network requires a greater effort to disseminate information across the entire network. This change suggests that during the COVID-19 pandemic, students tended to form smaller, tightly-knit communities, potentially concentrating information sharing and communications within these groups.

When we compared the alignment with the community members’ affiliation with the university (ie, their university name), we found that the communities identified by the friendship network were well aligned with the members’ actual university (or college) affiliations. The community members’ affiliation composition heterogeneity is calculated with the membership entropy to quantify the affiliation complexity (as referred in the Methods section). The peri–COVID-19 pandemic network demonstrates a smaller variance in *S* compared to the variance of *S* in the pre–COVID-19 pandemic network, as shown in the kernel density estimate plot in Figure 3C. The mean of the membership entropy 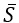 during the pre–COVID-19 pandemic period is approximately 1.0; this value during the peri–COVID-19 pandemic period is decreased to approximately 0.5. Because larger entropy means a large discrepancy between physical affiliation and community membership in internet-based social networks, the relatively small membership entropy during the peri–COVID-19 pandemic period demonstrates that a structurally identified community contains more homogeneous users in terms of their affiliated universities when compared with the pre–COVID-19 pandemic period.

### The Microview 2: Connection Patterns of the D-R and Non–D-R Groups Focusing on Filtered Friendship Networks of HBCU Students

**Figure 4:**
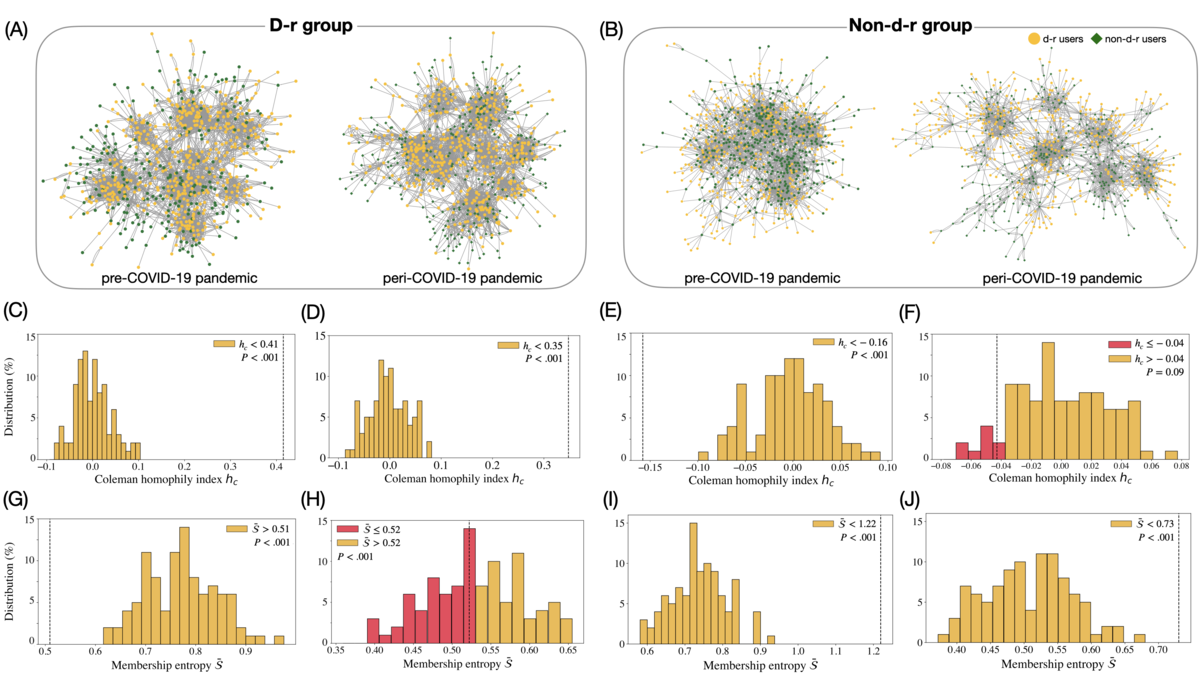
The depression-related (d-r) and non–depression related (non–d-r) groups’ friendship networks, composed of students from historically black colleges and universities before and during the COVID-19 pandemic. (A) The panel demonstrates the structural change of the d-r group’s friendship network in pre–COVID-19 (2019 dataset) and in peri–COVID-19 (2022 dataset) pandemic periods. (B) The panel displays the same structural changes of the non–d-r group’s friendship network in pre–COVID-19 (2019 dataset) and peri–COVID-19 (2022 dataset) pandemic periods. (C to F). The distribution of Coleman homophily index (*h_c_*) of randomized networks for the d-r group (C: 2019 and D: 2022) and for the non–d-r group (E: 2019 and F: 2022) are compared with the empirically observed *h_c_* of the d-r and non–d-r groups (dashed black line). *P* values are evaluated from the *h_c_* of random networks after the permutation tests. Highlighted bars in red represent the dataset having less the empirically observed *h_c_*. (G to J) The distribution of <inline-graphic xlink:href="jmir_v26i1e55531_fig9.png" mimetype="image" xlink:type="simple"/> of randomized networks for d-r group (G:2019, H:2022) and for non-d-r group (I:2019, J:2022). Dashed black lines represent the empirically observed <inline-graphic xlink:href="jmir_v26i1e55531_fig9.png" mimetype="image" xlink:type="simple"/>, and red bars denote the dataset having less <inline-graphic xlink:href="jmir_v26i1e55531_fig9.png" mimetype="image" xlink:type="simple"/> than the empirically observed one.

When we extracted the friendship networks of a group of HBCU students (ie, d-r or non–d-r groups), the distinct structural patterns and homophilic connections could be clearly observed (as seen in Figures 4A and 4B). The more segregated community patterns of the non–d-r group during the COVID-19 pandemic, which is observed in Figure 4B, are correlated with the increased modularity of the non–d-r group of users (from 0.67 during pre–COVID-19 pandemic period to 0.73 during peri–COVID-19 pandemic period), while d-r group’s modularity has not largely changed, as shown in Figure 4A (from 0.75 during pre–COVID-19 pandemic period to 0.76 during peri–COVID-19 pandemic period).

The mixing patterns of each group also exhibited different arrangements. The d-r group’s average Coleman homophily index *h_c_* = 0.41 in pre-COVID-19 pandemic has remained approximately the same with a slight decrease during the COVID-19 pandemic. To assess the significance of these changes in *h*_c_, we conducted the permutation test by randomizing users’ depression-related attributes to generate 100 sample networks that represent each group’s friendship networks during pre–COVID-19 and peri–COVID-19 pandemic periods (seen in the Methods section). Figure 4C displays the distribution of *h_c_* for the randomized friendship networks of the d-r users before COVID-19 pandemic (2019 dataset); Figure 4D shows these data for the d-r users during the COVID-19 pandemic (2022 dataset). As one can see, the homogeneity of the d-r group regarding the depression-related attribute is significantly different from the randomized results (*P*<.001). In short, this result emphasizes the d-r group’s collective connectivity patterns with other d-r group members.

By contrast, the non–d-r group’s homophily has increased from *h_c_*= –0.16 before the COVID-19 pandemic to *h_c_*= –0.04 during the COVID-19 pandemic. This means that the non–d-r group members connected more with other non–d-r group members during the pandemic, as shown in Figures 4E and 4F. In the pre–COVID-19 pandemic period, non–d-r users connected with different group members (negative *h_c_*); however, they were linked more with other members of the same group in the peri–COVID-19 pandemic period (as seen in Figures 4B, 4E, and 4F).

The d-r group’s average membership entropy 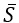 has been preserved during the COVID-19 pandemic from 0.51 to 0.52, as shown in Figures 4G and 4H, even though 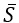 for the peri–COVID-19 pandemic period is statistically insignificant. In contrast, the non–d-r group demonstrated a statistically significant decrease in 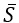 from 1.22 to 0.73 (as seen in Figures 4I and 4J). It made us infer that the non–d-r group’s communities are aligned more with users’ physical affiliations in the peri–COVID-19 pandemic period as compared with that in the pre–COVID-19 pandemic period (indicating low membership entropy). These results highlight the distinct internet-based social behaviors between the d-r and non–d-r groups in the course of the COVID-19 pandemic period. In other words, the d-r members’ communities maintained connections through shared physical affiliations during the COVID-19 pandemic period. These connections were different from the connection patterns of the non–d-r HBCU student group.

## Discussion

### Principal Findings

The COVID-19 pandemic profoundly impacted individuals, communities, economies, health care systems, and daily life worldwide [34-36]. The way individuals constructed social networks was no exception. Owing to the requirements of physical distancing, people use social media more than in the past to connect with others [12,13]. The increase in social media use on Twitter during the COVID-19 pandemic period was observed in both the d-r and non–d-r groups. However, the increase was statistically significant only in the d-r group. Moreover, users in the d-r group significantly increased mutual relationships (ie, reciprocity) and assortativity with other d-r users, whereas the non–d-r group’s reciprocity and assortativity decreased. The community detection and membership entropy analyses revealed an interesting pattern of internet-based socialization among HBCU students during the COVID-19 pandemic. The results show that the students strengthened their internet-based relationships on the basis of their physical affiliations in the course of the COVID-19 pandemic period, indicating localization of such socialization. The increase in agreement between internet-based relationships and offline affiliation is mostly observed in the non–d-r group. However, the d-r group has kept similar levels of connectivity during the COVID-19 pandemic, connecting with more d-r group of users in their internet-based community than the expectation from randomized networks. This study offers unique insights into the collective social networking patterns of young Black communities in HBCUs, with focus on depression-related expressions. This approach differs from traditional social relationship analysis conducted on the general population without providing information on the population group’s initial mental conditions [10,37].

### Distinct Internet-Based Socializing Patterns of the D-R Group

In this study, the increase in the number of friends per user during the COVID-19 pandemic for both groups may indicate a coping strategy of people, resulting in more activities on social media [12,13,37]. The internet has been a major source of health information related to the etiologies, interventions, and treatments for disease and illness [38]. Internet-based forms of social support also gained prominence and experienced substantial growth during the COVID-19 pandemic. This is because they allowed interactions with a broader network of people beyond geographic limitations, including the use and expansion of formal internet-based support spaces [10]. The statistically significant expansion of friendship shown only in the d-r group indicates a desire for greater connectedness in this group. This indirectly reflects a disproportional impact of the COVID-19 pandemic on people who experienced depression before the COVID-19 pandemic. This finding may inform higher education administrators and health organizations of the need for tailored mental wellness interventions for Black college students who mentioned depression on internet-based platforms.

In particular, the d-r group showed a statistically significant increase in reciprocity (mutual dependence) and assortativity (tendency to make friends in the same group) during the COVID-19 pandemic. In contrast, the non–d-r group experienced a decrease in both reciprocity (significant) and assortativity (not significant). The increased reciprocity and assortativity of the d-r group in an internet-based society might indicate that the tendency to associate with similar individuals became more prominent [10]; it might reflect that the d-r group pursued mutual exchange for information and social support, while the non–d-r group pursued more diverse information and social-support sources.

This distinct internet-based socializing pattern may bring several merits and benefits in different contexts, such as strong social bonds [27], fostered empathy [39], and efficient communication [40]. However, internet-based media can be a source of misinformation about health-related topics [40,41]. Considering the possibilities, we need to carefully understand the collective internet-based behaviors of a group of interest, especially of a minoritized group, which requires effort to understand their information consumption patterns [42]. Given the significant assortative tendency of the d-r group of users, identifying the d-r groups and designing a strategy to deliver accurate health information tailored for this group might be crucial to providing support during outbreaks such as the COVID-19 pandemic.

### Strengthening Physical Relationships via an Internet-Based Platform

On an average, the social networks of 2 HBCU groups showed that user interactions became more localized during the COVID-19 pandemic compared with that before the pandemic. When we focus solely on HBCU students, the results show more modular interactions that are densely connected between users with the same affiliations during the COVID-19 pandemic. The size of the communities becomes small, leading to increased modularity. The decreased membership entropy in the course of the COVID-19 pandemic indicates that people tend to make internet-based connections based on physical affiliations. Another study found evidence of network homophily, revealing that most teachers interacted primarily with colleagues from similar disciplines and the same country [43]. 

Even though the densely connected internet-based communities aligned with one’s physical affiliations can improve one’s connectedness and emergency support, information among the communities could often revolve around campus updates (eg, remote learning), local COVID-19 pandemic status updates (eg, vaccine availability), and connections within their immediate community [11,40]. These localized and assortative connections might influence the likelihood of exposure to reputable sources of information [44]. Strategies that are culturally appropriate, community competent, and mindful of the nuances of different populations and communities play a crucial role in promoting health equity [45]. The dissemination of health-related information, especially of minoritized groups on internet-based platforms, should be further explored.

However, the increase in Coleman homophily and modularity in the filtered HBCU students’ friendship network is mostly observed in non–d-r group of users than in the d-r group of users. This implies that the tendency to connect with local communities on the internet can be different by an individual’s engagement in depression-related content on the internet. The d-r group of users remained in a similar level of relational alignment with physical affiliations in their internet-based communities during the COVID-19 pandemic; the non–d-r group of users reached a comparable level of Coleman homophily and modularity to their counterparts in the course of the COVID-19 pandemic period. This result suggests that HBCU students with depression-related expressions before the pandemic primarily sought social connections from their preexisting interactions, unlike non-d-r users, who tended to seek more localized connections. Therefore, a different strategy may be needed to deliver appropriate health information to those with depression-related expressions.

As these results are shown, understanding a group’s social networks, risk experiences, and historical coping tendencies can help shape tailored strategies for health communication and treatment, as this information influences both internet-based and offline behavioral choices. In this study, insights from the internet-based socializing patterns of HBCU students can inform health care workers on therapeutic strategies tailored to patients within these communities. This might entail diversifying health-related information within their communities and nurturing sustainable localized communities with accurate health information and direct links to health care systems and support groups [42,43].

### Limitations

A limitation of this study is that Twitter does not allow easy filtering of users on the basis of demographics; therefore, verification of whether users meet the inclusion criteria was subjective and based on the available content in the user’s Twitter profile (eg, profile picture, account description, and tweets). This may exclude users who may have been eligible for inclusion. Because we could not reverify affiliation in the dataset published in 2022, the number of HBCU students in this study may be overestimated. In addition, because this study focuses on the social media activities and connections of the d-r group of users, the categorization of depression-related tweets cannot be used to confirm that the user has a clinical diagnosis of a depressive disorder. Therefore, the d-r group of users (or non–d-r group of users) could be overrepresented (or underrepresented) in the categorization.

### Conclusions

The results demonstrate that the social media activity of HBCU students with depression-related tweets can be significantly different from those without depression-related tweets. The d-r group of users tend to have more mutual relationships and try to connect with other d-r group of users. The HBCU students also strengthened their connections among themselves in the course of the pandemic. It is unclear whether having more friends in the same group can be positive (eg, social support) or negative (eg, harmful action) and how the information flow could differ from other demographic groups; thus, understanding these structural patterns related to the health-related information spreading dynamics could be an interesting future direction.

In addition, understanding minoritized groups’ internet-based social behaviors, such as that of Black American college students during the COVID-19 pandemic, can help to understand their social support–seeking behaviors during stressful times and inform the development of interventions to support their mental health. Future studies should further explore the relationship between social media and mental health to determine whether there is a pattern of increased social media activity, mental health information seeking, and social-support seeking in different periods of time during the year for Black college students.

## Abbreviations

d-r: depression-related
HBCU: historically black colleges and universities
LCC: largest connected component
non–d-r: non–depression-related

## Data Availability

The datasets collected during and analyzed during this study are not publicly available due to ethical considerations, since sharing the data may raise the possibility of revealing the identities of the Twitter users included in this study, which could put individuals at risk for harassment and discrimination.

## References

[1] Huckins JF, daSilva AW, Wang W, Hedlund E, Rogers C, Nepal SK, Wu J, Obuchi M, Murphy EI, Meyer ML, Wagner DD, Holtzheimer PE, Campbell AT (2020). Mental health and behavior of college students during the early phases of the COVID-19 pandemic: longitudinal smartphone and ecological momentary assessment study. J Med Internet Res.

[2] Charles NE, Strong SJ, Burns LC, Bullerjahn MR, Serafine KM (2021). Increased mood disorder symptoms, perceived stress, and alcohol use among college students during the COVID-19 pandemic. Psychiatry Res.

[3] Alcendor DJ (2020). Racial disparities-associated COVID-19 mortality among minority populations in the US. J Clin Med.

[4] Magesh S, John D, Li WT, Li Y, Mattingly-App A, Jain S, Chang EY, Ongkeko WM (2021). Disparities in COVID-19 outcomes by race, ethnicity, and socioeconomic status: a systematic-review and meta-analysis. JAMA Netw Open.

[5] Cunningham TJ, Croft JB, Liu Y, Lu H, Eke PI, Giles WH (2017). Vital signs: racial disparities in age-specific mortality among Blacks or African Americans - United States, 1999-2015. MMWR Morb Mortal Wkly Rep.

[6] Millett GA, Jones AT, Benkeser D, Baral S, Mercer L, Beyrer C, Honermann B, Lankiewicz E, Mena L, Crowley JS, Sherwood J, Sullivan PS (2020). Assessing differential impacts of COVID-19 on Black communities. Ann Epidemiol.

[7] Olaniyan M, Magnelia S, Coca V, Abeyta M, Vasquez MC, Harris F III, Gadwah-Meaden C (2023). Two pandemics: racial disparities in basic needs insecurity among college students during the COVID-19 pandemic. The Hope Center.

[8] Mccall T, Kim H, Lee E, Lakdawala A, Bolton Iii CS (2021). Content and social network analyses of depression-related tweets of African American college students. Proceedings of the 54th Hawaii International Conference on System Sciences.

[9] Higher Education Act of 1965. U.S. Government Publishing Office.

[10] Long E, Patterson S, Maxwell K, Blake C, Bosó Pérez R, Lewis R, McCann M, Riddell J, Skivington K, Wilson-Lowe R, Mitchell KR (2022). COVID-19 pandemic and its impact on social relationships and health. J Epidemiol Community Health.

[11] (2021). A look at volunteering during the response to COVID-19. Department for Digital, Culture, Media & Sport, United Kingdom Government.

[12] Pandya A, Lodha P (2021). Social connectedness, excessive screen time during COVID-19 and mental health: a review of current evidence. Front Hum Dyn.

[13] Juvonen J, Schacter HL, Lessard LM (2021). Connecting electronically with friends to cope with isolation during COVID-19 pandemic. J Soc Pers Relatsh.

[14] Stuart J, O'Donnell K, O'Donnell A, Scott R, Barber B (2021). Online social connection as a buffer of health anxiety and isolation during COVID-19. Cyberpsychol Behav Soc Netw.

[15] Rauschenberg C, Schick A, Goetzl C, Roehr S, Riedel-Heller SG, Koppe G, Durstewitz D, Krumm S, Reininghaus U (2021). Social isolation, mental health, and use of digital interventions in youth during the COVID-19 pandemic: a nationally representative survey. Eur Psychiatry.

[16] Gabbiadini A, Baldissarri C, Durante F, Valtorta RR, De Rosa M, Gallucci M (2020). Together apart: the mitigating role of digital communication technologies on negative affect during the COVID-19 outbreak in Italy. Front Psychol.

[17] Li J (2023). Digital technologies for mental health improvements in the COVID-19 pandemic: a scoping review. BMC Public Health.

[18] (2020). Stress in America™ 2020: a national mental health crisis. American Psychological Association.

[19] Provisional death counts for COVID-19. Centers for Disease Control and Prevention.

[20] Kemei J, Tulli M, Olanlesi-Aliu A, Tunde-Byass M, Salami B (2023). Impact of the COVID-19 pandemic on Black communities in Canada. Int J Environ Res Public Health.

[21] Yancy CW (2020). COVID-19 and African Americans. JAMA.

[22] Mercier CM, Abbott DM, Ternes MS (2022). Coping matters: an examination of coping among Black Americans during COVID-19. Couns Psychol.

[23] Molock SD, Parchem B (2022). The impact of COVID-19 on college students from communities of color. J Am Coll Health.

[24] Ramos MS, Corona R, Dempster KW, Morton SC, Everhart RS (2023). The COVID-19 pandemic: asthma control, tobacco use, and mental health among African American and Latinx college students. J Asthma.

[25] Huang HY, Li H, Hsu YC (2022). Coping, COVID knowledge, communication, and HBCU student's emotional well-being: mediating role of perceived control and social connectedness. J Community Psychol.

[26] Fetter A, Thompson M (2020). Understanding the impacts of COVID-19 pandemic for undergraduate students attending an HBCU: insights from student voices. Center for Research on College-Workforce Transitions, Wisconsin Center for Education Research.

[27] Perry BL, Smith NC, Coleman ME, Pescosolido BA (2024). Social networks, the COVID-19 pandemic, and emerging adults' mental health: resiliency through social bonding and cohesion. Am J Public Health.

[28] Moreno JL, Jennings HH (1938). Statistics of social configurations. Sociometry.

[29] Newman M (2018). Networks.

[30] Moody J (2001). Race, school integration, and friendship segregation in America. Am J Sociol.

[31] Blondel VD, Guillaume JL, Lambiotte R, Lefebvre E (2008). Fast unfolding of communities in large networks. J Stat Mech Theor Exp.

[32] Coleman J (1958). Relational analysis: the study of social organizations with survey methods. Hum Org.

[33] Newman ME (2006). Modularity and community structure in networks. Proc Natl Acad Sci U S A.

[34] (2020). Global state of small business report. Facebook, OECD, World Bank.

[35] Reinhart CM (2021). From health crisis to financial distress. Policy research working paper. World Bank.

[36] Haleem A, Javaid M, Vaishya R (2020). Effects of COVID-19 pandemic in daily life. Curr Med Res Pract.

[37] Kovacs B, Caplan N, Grob S, King M (2021). Social networks and loneliness during the COVID-19 pandemic. Socius.

[38] National Research Council, Commission on Physical Sciences, Mathematics, and Applications, Computer Science and Telecommunications Board, Committee on Enhancing the Internet for Health Applications: Technical Requirements and Implementation Strategies (2000). Networking Health: Prescriptions for the Internet.

[39] Chen Y, Xu Y (2021). Exploring the effect of social support and empathy on user engagement in online mental health communities. Int J Environ Res Public Health.

[40] Baker H, Concannon S, So E (2022). Information sharing practices during the COVID-19 pandemic: a case study about face masks. PLoS One.

[41] Cuan-Baltazar JY, Muñoz-Perez MJ, Robledo-Vega C, Pérez-Zepeda MF, Soto-Vega E (2020). Misinformation of COVID-19 on the internet: infodemiology study. JMIR Public Health Surveill.

[42] Reddy B, Gupta A (2020). Importance of effective communication during COVID-19 infodemic. J Family Med Prim Care.

[43] Alwafi E (2021). Tracing changes in teachers' professional learning network on Twitter: comparison of teachers' social network structure and content of interaction before and during the COVID-19 pandemic. J Comput Assist Learn.

[44] Hâncean MG, Lerner J, Perc M, Molina JL, Geantă M (2024). Assortative mixing of opinions about COVID-19 vaccination in personal networks. Sci Rep.

[45] Webb Hooper M, Nápoles AM, Pérez-Stable EJ (2020). COVID-19 and racial/ethnic disparities. JAMA.

